# Chromatin insulation dynamics in glioblastoma: challenges and future perspectives of precision oncology

**DOI:** 10.1186/s13148-021-01139-w

**Published:** 2021-07-31

**Authors:** Borja Sesé, Miquel Ensenyat-Mendez, Sandra Iñiguez, Pere Llinàs-Arias, Diego M. Marzese

**Affiliations:** Cancer Epigenetics Laboratory At the Cancer Cell Biology Group, Institut D’Investigació Sanitària Illes Balears (IdISBa), Carretera de Valldemosa 79, S Building, 1st Floor, 07120 Palma de Mallorca, Spain

## Abstract

Glioblastoma (GBM) is the most aggressive primary brain tumor, having a poor prognosis and a median overall survival of less than two years. Over the last decade, numerous findings regarding the distinct molecular and genetic profiles of GBM have led to the emergence of several therapeutic approaches. Unfortunately, none of them has proven to be effective against GBM progression and recurrence. Epigenetic mechanisms underlying GBM tumor biology, including histone modifications, DNA methylation, and chromatin architecture, have become an attractive target for novel drug discovery strategies. Alterations on chromatin insulator elements (IEs) might lead to aberrant chromatin remodeling via DNA loop formation, causing oncogene reactivation in several types of cancer, including GBM. Importantly, it is shown that mutations affecting the isocitrate dehydrogenase (IDH) 1 and 2 genes, one of the most frequent genetic alterations in gliomas, lead to genome-wide DNA hypermethylation and the consequent IE dysfunction. The relevance of IEs has also been observed in a small population of cancer stem cells known as glioma stem cells (GSCs), which are thought to participate in GBM tumor initiation and drug resistance. Recent studies revealed that epigenomic alterations, specifically chromatin insulation and DNA loop formation, play a crucial role in establishing and maintaining the GSC transcriptional program. This review focuses on the relevance of IEs in GBM biology and their implementation as a potential theranostic target to stratify GBM patients and develop novel therapeutic approaches. We will also discuss the state-of-the-art emerging technologies using big data analysis and how they will settle the bases on future diagnosis and treatment strategies in GBM patients.

## Introduction

Glioblastoma (GBM) is the most aggressive type of primary brain tumor. The current standard-of-care (SOC) for patients with GBM includes a combination of surgical resection, adjuvant radiotherapy, and chemotherapy, mainly based on temozolomide (TMZ) [1, 2]. However, the prognosis of GBM patients remains dismal, with a median survival time of approximately 15 months and a recurrence rate of about 90% [3]. In addition to the limited benefit in survival, SOC treatments cause significant morbidity involving neurological deficits. Formerly known as glioblastoma multiforme, the term “multiforme” reflects a robust heterogeneous variety of cell types coexisting within the tumor. Each cell type exhibits a particular molecular profile, leading to different degrees of therapy resistance among its tumor cell population [4, 5]. The detection and characterization of such intratumor heterogeneity are of great value to the clinical diagnosis and management of this disease. GBM can develop rapidly as a de novo brain tumor (primary GBM) in more than 90% of cases [6]. To a lesser extent, these tumors can originate from previous lower-grade diffuse gliomas (secondary GBM). Although these are histologically indistinguishable, they present distinct genetic and epigenetic signatures that allow their identification.

Recent molecular and computational biology improvements allowed the identification of novel targetable molecular mechanisms in GBM. Gene- and gene pathway-centered approaches have generated a myriad of data about GBM mechanisms contributing to invasion, progression, unlimited replication, maintenance, and drug resistance [7–9]. However, to date, the contribution of these scientific advances to the clinical management of GBM patients remains insufficient. The limited improvements in the clinical outcomes reflect the inherent multi-molecular-level, omics-scale complexity that defines GBM etiology and pathology. The absence of effective therapeutic management represents an inherent challenge to treat GBM. Taken together, these issues motivate the need for alternative approaches to better understand and disentangle the integrative molecular alterations underpinning the aggressive and treatment-resistant phenotype of GBM.

Genetic and epigenetic alterations on insulator elements (IEs), an essential type of *cis*-regulatory element involved in enhancer–promoter interactions, have been recently found in cancer cells [10]. In particular, dysfunctional IEs result in aberrant chromatin conformation and the consequent oncogene activation [11]. Therefore, this review covers the most recent findings on the role of IEs in GBM and the potential effects on gene network regulation. At the same time, it provides an overview of the chromatin architecture and IEs in glioma stem cells (GSCs) and its potential translation into novel patient-centered diagnosis, prognosis, and therapeutic applications.

### Role of insulator elements in chromatin organization, gene isolation, and concomitant gene expression

The essential role of IEs is the compartmentalization of chromatin into structural and functional units known as Topologically Associating Domains (TADs). TADs are megabase-size regions formed and maintained by the architectural protein CCCTC-binding factor (CTCF) that restrict chromatin interactions within themselves compared to neighboring genomic regions [12]. TADs can act as a single insulated region or as a multidomain structure containing several insulated segments, often referred to as sub-TADs. Both TADs and sub-TADs are organized into distinct units of globular conformation, forming DNA loops that confer physical isolation of genes confined within a TAD structure that prevents their interaction with regulatory regions located outside of the TAD [12, 13]. It has been proposed that TAD formation follows the “loop extrusion” model where CTCF and cohesin are the major players [14–17]. In this model, the cohesin complex translocates onto chromatin. It travels along the DNA molecule, extruding a chromatin loop on its way until it reaches a CTCF bound to an inward-oriented CTCF site, blocking further chromatin extrusion.

**Fig. 1:**
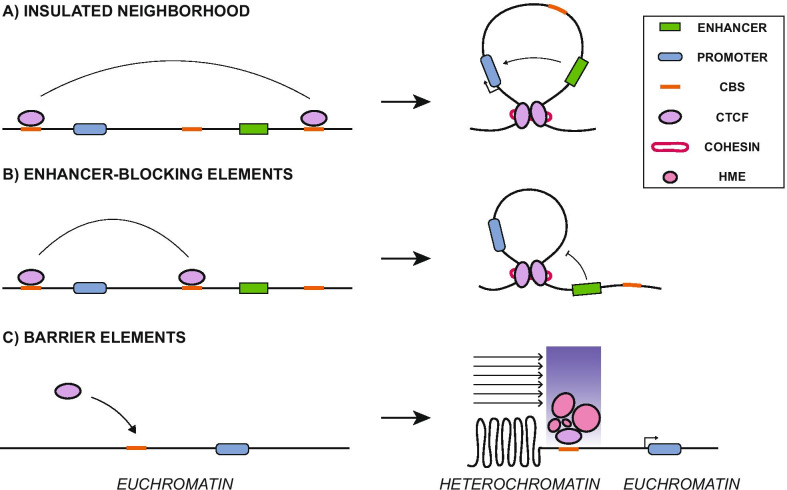
Insulator elements mechanism of action. **a** Insulator flanking a transgene and an enhancer element generates an insulated neighborhood via CTCF-loop formation, favoring enhancer–promoter interactions within the DNA loop. **b** Enhancer-blocking elements result from insulators between a transgene and an enhancer, leaving the enhancer outside the DNA loop, blocking its interaction with the transgene promoter. **c** Insulators acting as barrier elements prevent the spread of heterochromatin by recruiting histone-modifying enzymes (HME) to preserve a transcriptionally active euchromatin state

IEs regulate gene expression via loop formation in a position-dependent manner [18]. CTCF-cohesin anchored loops can facilitate enhancer–promoter interactions when flanking both elements to constrain the enhancer function within the DNA loop, resulting in a structural unit known as an “insulated neighborhood” [19] (Fig. 1a). Conversely, IEs can also block enhancer–promoter interactions by capturing a gene promoter inside a chromatin loop, unable to reach its former enhancer element (EE). This type of IE is known as “enhancer-blocking elements” and can only occur if the insulator sequence is placed between the EE and the gene promoter (Fig. 1b). Also, IEs can separate active chromatin (euchromatin) from repressive chromatin regions (heterochromatin), independently of CTCF-loop formation. This latter type of IE is known as “barrier elements,” and their mechanism of action consists of recruiting histone modifying enzymes, acting as a physical barrier to protect against the linear spread of heterochromatin into euchromatin regions, and vice versa [20] (Fig. 1c). All these IE functions are notably important in sub-TADs, where they grant a more dynamic regulatory control over genes located within the loop structure [21]. While TADs are highly conserved among mammals and different tissues, sub-TADs are more variable. They tend to be associated with changes in response to cellular signals, gene activity and allow for cell-specific functions [22, 23]. These observations suggest that sub-TADs allow fine-tuned control of the gene expression during specific transcriptional programs such as cell differentiation [24].

### Aberrant chromatin insulation function in cancer

Alterations in TAD assembly and high-order chromatin organization are often associated with multiple developmental defects and diseases. While many efforts have been made to study transcriptional regulatory elements in cancer, most of these focused on promoters and EEs. Regarding IEs, loss of CTCF boundaries may disrupt insulated neighborhoods containing oncogenes, thus becoming reactivated [25]. Alternatively, alterations in gene expression could influence and reshape chromatin loops to gain tumor-related traits, as shown in several types of cancer [26]. Aberrant insulator function could be associated with missense mutations in the CTCF coding region and CTCF binding sites (CBSs), leading to disrupted loop-formation activity and gene expression dysregulation [27–29]. Supporting the relevance of chromatin insulation in cancer, Liu et al. identified 21 IE mutations as putative drivers of multiple cancer types [10]. In addition to somatic mutations, CBSs could also be affected by epigenetic modifications. The hypomethylation of CBS promotes a higher occupancy by CTCF, resulting in de novo formation of TAD boundaries causing deregulation of gene expression often linked to tumor suppressor silencing [30]. On the contrary, hypermethylation of CBS reduces the recruitment of CTCF, which is followed by the disruption of TAD domains and, in many cases, leads to aberrant promoter–enhancer interactions known as “enhancer adoption” [31]. This enhancer adoption event is frequently related to oncogene activation [32].

### Dynamic function of chromatin insulation in glioblastoma

Given GBM intratumor heterogeneity, a significant effort has been made to search for epigenetic features to stratify these tumors into subcategories for accurate diagnosis and treatment [33, 34]. The major genomic alterations described in GBM are the mutations affecting the isocitrate dehydrogenase (IDH) 1 and 2 genes. IDH enzymes are best known for playing an essential role in several metabolic processes. As such, recurrent mutations of these genes are present in various human malignancies [35]. While IDH wild-type (IDH-wt) enzymes convert isocitrate into α-ketoglutarate (α-KG) as part of the Krebs cycle, IDH mutant (IDH-mt) enzymes lose their affinity for isocitrate and convert α-KG into D-2-hydroxyglutarate (D-2-HG) [36]. Accumulation of D-2-HG leads to severe epigenetic alterations by blocking the activity of ten-eleven translocation methylcytosine dioxygenase (TET), a family of enzymes involved in DNA demethylation [37]. Consequently, IDH mutations result in widespread CpG island methylation, a particular signature known as glioma CpG island methylator phenotype (G-CIMP). Given the dramatic clinical and molecular differences associated with the G-CIMP, GBM is classified into two distinctive categories: IDH-mt and IDH-wt [38]. IDH-mt GBMs represent less than 10% of GBM cases, frequently associated with younger individuals, and typically correspond with secondary GBM. On the other hand, IDH-wt GBMs comprise most GBM cases, mostly primary tumors, often diagnosed in elderly patients with a significantly worse prognosis than IDH-mt GBM [39]. Inevitably, genome-wide DNA hypermethylation has a notorious impact on chromatin insulation by compromising IE functionality. Flavahan et al. [11] reported that many CTCF sites were lost in IDH-mt GBM. A well-studied case of insulator dysfunction in IDH-mt occurs after hypermethylation of CBS and the consequent disruption of TAD boundaries, enabling the interaction of a constitutive EE with the promoter region of the receptor tyrosine kinase platelet-derived growth factor receptor A (*PDGFRA*) gene (enhancer adoption), a well-described glioma oncogene [11]. However, most likely, many other oncogenic events in GBM are deregulated due to epigenetic alterations and chromatin insulation loss. Expanding our understanding of how IEs reshape chromatin and lead to oncogenic reactivation in GBM may uncover novel therapeutic targets to improve the diagnosis and targeted treatments.

### Insulator elements influence gene expression in glioma stem cells

Malignant tumors consist of heterogeneous cell populations, including a small subset called cancer stem cells (CSCs), or tumor-initiating cells, which are thought to initiate tumor formation, promote metastasis, and grant resistance to therapy [40–42]. In GBM, these cells are known as GSCs and have become a key target for therapeutic developments with the aim to eliminate tumor therapy-resistant traits and relapse [43–46]. GSCs must activate several signaling pathways such as NOTCH, bone morphogenetic protein (BMP), wingless-related integration site (WNT), epidermal growth factor (EGF), and sonic hedgehog (SHH) to maintain an undifferentiated state often associated with stemness and required for self-renewal and rapid differentiation upon tumor progression in CSCs [47]. This provides GSCs a cellular plasticity capable of generating all the different cell types found in the tumor bulk. Also, GSCs present genomic regions where active histone marks (H3K4me3) coexist with repressive histone modifications (H3K27me3), the so-called bivalent domains, often associated with repressed lineage-specific genes to maintain an undifferentiated state [48, 49]. A recent work published by *Hall* et al*.* [49] has shown that bivalent regions within GBM primary tumors are part of a highly interconnected network under the influence of WNT, SHH, and HOX pathways, commonly associated with embryonic development. Thus, a subset of transcription factors (TFs) may be responsible for establishing a permissive chromatin architecture that maintains stemness through several cell divisions in GSCs, which, in turn, confers aggressive traits, including tumor progression and drug resistance.

A proper chromatin assembly into structural subunits is required to coordinate specific gene expression programs to establish and maintain GSC stemness. GSCs present a specific subset of large clusters of EEs known as super-enhancers (SEs) that drive a robust transcriptional program determined by core TFs [50]. A recent study conducted by Johnston et al*.* [51] revealed that genes interacting with SEs within a DNA loop are highly expressed in GSCs. Moreover, some of these loops containing SEs seem to be GSC-specific as they are strongly conserved among different GSC lines. In this same work, the authors also showed that structural variants in the GSC genome cause rare long-distance loops resulting in de novo SE-promoter interactions. Most of these gene sets, highly connected through extensive chromatin looping, play a significant role in brain tumors and stem cell biology. Also, an enrichment of TFs regulated by GSC-specific SEs is associated with shorter survival of GBM patients, suggesting an essential role of SEs mediating the transcriptional regulatory program behind the maintenance of a GSC phenotype [50]. These data highlight the importance of IEs and TAD formation as a key regulatory process to assemble the GSC epigenome.

**Fig. 2:**
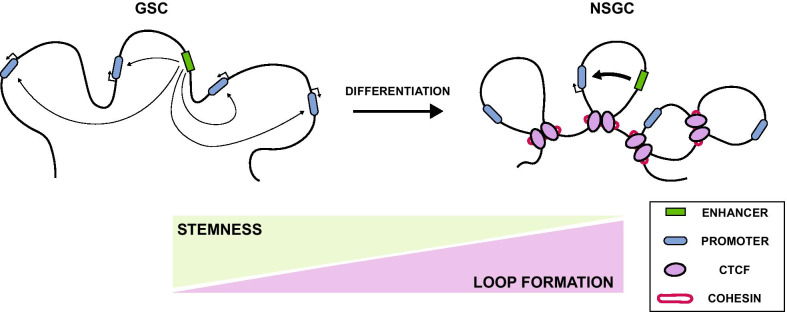
Chromatin loop formation in GSC differentiation. GSCs present a more relaxed and accessible chromatin state with permissive interactions between enhancers and promoters among different domains. During GSC differentiation, loss of stemness in non-stem glioma cells (NSGC) correlates with increased chromatin-loop formation, adopting a more restricted chromatin conformation with strong domain boundaries and limited enhancer–promoter interactions

Genome architecture and chromatin insulation are essential to maintain cells in an undifferentiated state, propitiate cell plasticity, and coordinate differentiation programs into cell lineages. Differentiation of embryonic stem cells (ESCs) into neural precursors has been correlated with a gain of structural loops and enhanced binding of CTCF and cohesin leading to durable insulation between chromatin boundaries, limiting the enhancer–promoter interaction to the detriment of the activation of developmental genes [52]. Similar to ESCs, GSCs also exhibit a more accessible genome-wide chromatin state with a global loss of repressive (H3K9me3) and gain of active (H3K9ac) histone modification marks [53]. Based on these findings, GSCs may present a similar behavior to ESCs, where a permissive chromatin state is required to sustain self-renewal, and the structural loop rearrangements are needed to reinforce chromatin boundaries and give rise to differentiated progeny that will constitute the tumor cell mass (Fig. 2). For example, a neural stem cell model for low-grade astrocytoma containing *IDH1/2* gene mutations caused the loss of CTCF binding and the subsequent dissociation of the sex determining region y-box 2 (*SOX2*) promoter–enhancer interaction [54]. This alteration resulted in an impaired ability to differentiate in vivo due to chromatin loop disruption, thus increasing its self-renewal and invasiveness capacities. Taken together, IEs could emerge as a new therapeutic target to prevent tumor growth in GBM. However, further analyses are required to elucidate the impact of IEs and chromatin architecture in GSCs on drug resistance, tumor survival, and relative toxicity and off-target effects of its inhibition.

### How to modulate epigenomic alterations in glioblastoma? Technology for druggability testing

GBM treatment remains a challenge as current therapies can only improve median prognosis by a few months. Epigenetic modulators have become an attractive therapeutic target for drug discovery to efficiently regulate cancer growth and progression. Numerous studies on histone deacetylases inhibitors have shown promising results in various cancers [55, 56], including GBM [57–61]. Following this research line, there is a particular interest in identifying novel compounds to target cancer regulatory elements. The JQ1, a bromodomain and extra-terminal domain (BET) domain inhibitor, has been shown to downregulate the expression of well-known oncogenes such as the MYC proto-oncogene, bHLH transcription factor (*MYC*) and B-cell lymphoma 2 (*BLC2*) by blocking bromodomain-containing protein 4 (BRD4) binding at associated SE regions in different cancers [62, 63]. Combined with conventional chemotherapy agents, BET inhibitors showed a significant anti-tumor effect in GBM xenograft models [64]. However, the efficacy of BET inhibitors in clinical trials remains untested. Recent studies have revealed the importance of architectural proteins in cancer treatment and how aberrant CTCF function can contribute to a drug-resistant phenotype [26, 65]. In a recent work, Kantidze et al. discovered curaxins, a novel class of DNA intercalating agents that promote the dissociation of CTCF from its binding site causing chromatin loop disorganization in tumor cells [66, 67]. These modifications resulted in transcriptional suppression of several MYC family oncogenes after losing their long-distance interactions with their respective SEs. These findings support the potential of epigenetic therapies targeting GBM-specific IEs involved in tumorigenesis and therapy resistance. This strategy is currently being tested in phase I clinical trial for patients with cutaneous melanoma or sarcoma (ClinicalTrials.gov, NCT03727789). Nevertheless, the implementation of this strategy is still non-genome site specific. A novel clustered regularly interspaced short palindromic repeats (CRISPR)-based approach consisting of a catalytically inactive CRISPR-associated protein 9 (dCas9) fused with the Krüppel-associated box (KRAB) transcriptional repressor and DNA-methyltransferase 3A (DNMT3A) can selectively disrupt CTCF recruitment to its CBS, allowing to study the role of IEs in a locus-specific manner [68]. Although Cas9-fused proteins are far from being used to treat GBM, the first-ever clinical trial to directly deliver CRISPR-Cas9 in the body has recently begun recruiting patients with retinal diseases (ClinicalTrials.gov, NCT03872479), bringing this technology one step closer for clinical applications. Forward-thinking, a potential strategy would involve restoring chromatin conformation by targeting IEs involved in enhancer–promoter interactions to prevent (1) oncogene activation, (2) tumor suppressor gene silencing, and (3) interaction of SEs with stemness genes in GSCs. However, despite the promising potential of drugs targeting tumor cells genome-wide organization, further studies would be required to anticipate and prevent undesired outcomes. Therefore, a better understanding of chromatin insulators and their role in dynamic chromatin organization is a crucial initial step toward a more effective therapeutic combination and personalized anti-GBM therapy.

### Impact of insulator elements on glioblastoma precision oncology

**Fig. 3:**
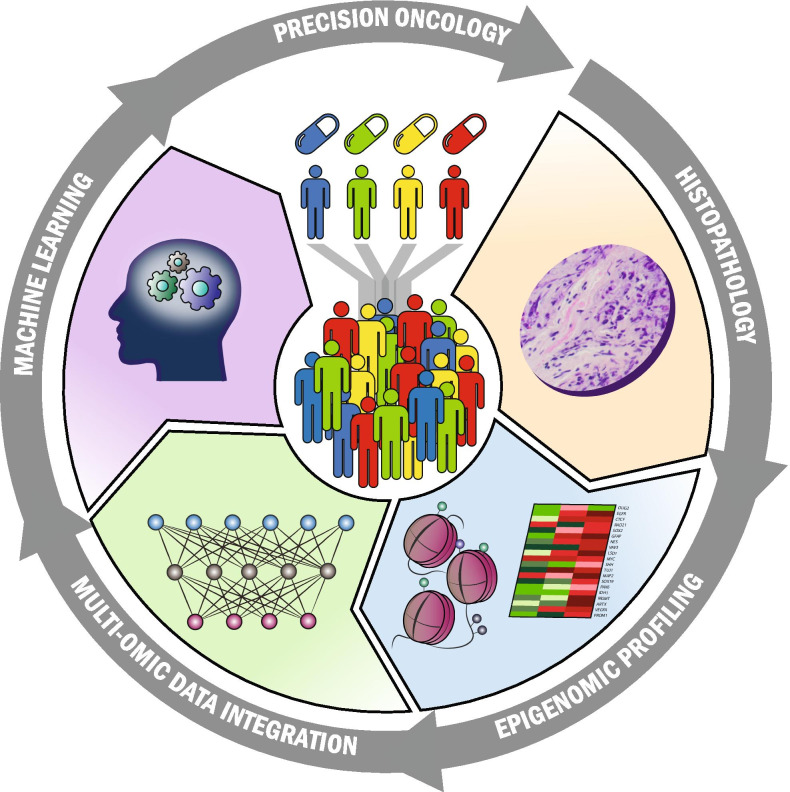
Precision neuro-oncology cycle. Patient stratification based on traditional histopathological evaluation of GBM tumors improves by implementing genome-scale epigenomic profiling, multidimensional data integration, and identifying the minimum and highly informative nomograms using Artificial Intelligence and Machine learning techniques

The next frontier in the clinical management of GBM is the implementation of precision medicine models to generate biomarkers for early detection, identification of patient-tailored treatments, and personalized follow-up. Advances in GBM epigenetics, particularly IEs modulating chromatin conformation, could provide additional information on current multi-omics profiling. Epigenetic alterations in GBM are currently employed for response to TMZ prediction (O-6-methylguanine-DNA methyltransferase (*MGMT*) gene promoter hypermethylation) [69], prognosis estimation (G-CIMP) [70], and molecular stratification (GBM molecular subtype classification) [5]. Advances in integrating the patient’s and tumor’s genetic makeups with epigenome-wide DNA methylation profiles, chromatin conformation, and TF occupancy to generate gene regulatory circuits active in each patient will improve our ability to stratify patients and improve clinical management (Fig. 3). These changes will advance our ability to build accurate models capable of determining the best available treatment and improve the clinical management of this deadly disease. As recently proposed, the implementation of systems biology approaches to integrate clinical with multi-omics data, including epigenetics, provides the basis to design *N-of-1* precision medicine treatments [71]. Examples of these advances involve the repurposing of drugs and treatments that have shown poor results in unselected cohorts of GBM patients. Such an endeavor will be appeased by emerging technologies capable of profiling epigenetic landscapes with minimum tissue requirements. The most remarkable improvements in this area involve the capabilities to perform dual chromatin accessibility and DNA methylation profiling [72, 73], long-read sequencing that allows profiling DNA modifications beyond 5-methylcytosine, and chromatin accessibility [72, 74–76], and its applications to single-cell samples.

Regarding the study of chromatin architecture, many techniques have been developed during the last years. 3D chromatin is mainly assessed through three different approaches: (1) by imaging, which includes DNA-Fluorescence in Situ Hybridization (DNA-FISH) and its derivatives; (2) using chromatin conformation capture (3C)-derived techniques, which require the ligation of DNA fragments; and (3) performing ligation-free methods. The advantages and limitations of these techniques are greatly discussed in a recent review by Kempfer et al. [77]. The rise of the studies of 3D chromatin may increase our understanding of GBM biology. However, many of the current techniques require large amounts of cells, representing an unsolved limitation. To overcome this restriction, the techniques that may be translated to the bedside for GBM are genome architecture mapping (GAM) and Capture Hi-C, which require less than 10^5^ cells. GAM is based on laser microdissection on sucrose-embedded cells, followed by DNA sequencing [78]. Capture Hi-C is a 3C-based method where the 3C library can be enriched for targets of interest. This approach has been used to detect interactions to promoter regions genome-wide [79], but it can also be performed to detect interactions between IEs.

On the other hand, genetic variations affecting non-coding genomic regions are starting to find functional relevance due to the integration of Genome-Wide Association Studies (GWAS) datasets with GBM epigenetic profiles [80]. For example, mutations in the promoter region of the *TERT* gene modify the binding affinity of the cAMP response element-binding protein (CREB) transcription factor and lead to an enhanced expression of the telomerase gene in several types of cancer [81, 82]. Today, this non-coding alteration is part of the genetic changes considered for GBM classification [83]. Similarly, we believe that dysfunctional IEs could be considered potential theranostic markers for personalized treatments in GBM. A significant milestone to successfully apply this strategy is to comprehensively overlay mutation signatures, gene expression, and clinical information with master TF regulators of the epigenomic dynamics. Thus, key TFs and microRNA orchestrating transcriptional regulatory networks (TRN) in gliomas have been unraveled thanks to the development and application of a platform known as the GBM SYstems Genetics Network AnaLysis pipeline (gbmSYGNAL) [84]. This pipeline has shown that TRN structures can be integrated with data from currently-approved cancer drugs to identify patient-specific novel and synergistic therapeutic interactions. This pioneering development is just the start of the upcoming era of GBM precision medicine aided by epigenomic networks.

## Conclusions

Although TAD formation and its role in regulating gene expression have been widely studied over the last years, the mechanisms on how enhancers preferentially interact with their target genes are still not fully understood. As a result, alterations of non-coding regulatory elements associated with tumorigenesis, and IEs in particular, have been largely underestimated. Given the relevance of these high-order chromatin alterations in driving oncogenic programs, continued efforts to uncover IE regulatory mechanisms are required. In GBM, an in-depth look at how IEs reshape chromatin topology to achieve a malignant phenotype will be paramount to find new therapeutic strategies against this devastating type of tumor. The road to integrating epigenomics into clinical decision-making algorithms for patients with GBM will be paved with progress in computing capabilities and enhanced analytical algorithms. Technological advances are needed to reduce the complexity of widespread aberrant chromatin insulation and its impact on long-range interactions among genomic regulatory elements. Computer science leading the incorporation of artificial intelligence into clinical nomograms to generate integrative systems for medicine is changing our approaches to analyze large-scale and complex datasets. Machine learning algorithms are already changing the field of cancer diagnosis and prognosis by exploring diverse data types, including imaging, histology, and multi-omics, to efficiently classify various clinically relevant GBM traits [85–88]. These advances will undoubtedly bring novel and more personalized diagnostic and therapeutic alternatives for patients with GBM.

## Data Availability

Not applicable.

## Abbreviations

GBM: Glioblastoma
IE: Insulator element
IDH: Isocitrate dehydrogenase
IDH-wt: Isocitrate dehydrogenase wild-type
IDH-mt: Isocitrate dehydrogenase mutant
GSC: Glioma stem cells
SOC: Standard-of-care
TMZ: Temozolomide
TAD: Topologically Associating Domains
CTCF: CCCTC-binding factor
EE: Enhancer element
CBS: CTCF binding sites
α-KG: α-Ketoglutarate
D-2-HG: D-2-hydroxyglutarate
TET: Ten-eleven translocation methylcytosine dioxygenase
G-CIMP: Glioma CpG island methylator phenotype
PDGFRA: Platelet-derived growth factor receptor A
CSC: Cancer stem cell
BMP: Bone morphogenetic protein
WNT: Wingless-related integration site
EGF: Epidermal growth factor
SHH: Sonic hedgehog
H3K4me3: Histone H3 lysine K4 trimethylation
H3K27me3: Histone H3 lysine K27 trimethylation
H3K9me3: Histone H3 lysine K9 trimethylation
H3K9ac: Histone H3 lysine K9 acetylation
TF: Transcription factor
SE: Super-enhancer
ESC: Embryonic stem cell
SOX2: Sex-determining region y-box 2
BET: Bromodomain and extraterminal
BLC2: B-cell lymphoma 2
BRD4: Bromodomain-containing protein 4
MYC: MYC proto-oncogene, bHLH transcription factor
CRISPR: Clustered regularly interspaced short palindromic repeats
Cas9: CRISPR-associated protein 9
KRAB: Krüppel-associated box
DNMT3A: DNA-methyltransferase 3A
MGMT: O-6-methylguanine-DNA methyltransferase
3D: Three-dimensional
DNA-FISH: DNA-fluorescence in situ hybridization
GAM: Genome architecture mapping
3C: Chromosome conformation capture
Hi-C: High-throughput chromosome conformation capture
TERT: Telomerase reverse transcriptase
CREB: CAMP response element-binding
TRN: Transcriptional regulatory networks
gbmSYGNAL: Glioblastoma systems genetics network analysis pipeline
HME: Histone-modifying enzymes
NSGC: Non-stem glioma cells
