# Correction to: Chromatin insulation dynamics in glioblastoma: challenges and future perspectives of precision oncology

**DOI:** 10.1186/s13148-021-01185-4

**Published:** 2021-10-19

**Authors:** Borja Sesé, Miquel Ensenyat-Mendez, Sandra Iñiguez, Pere Llinàs-Arias, Diego M. Marzese

**Affiliations:** Cancer Epigenetics Laboratory At the Cancer Cell Biology Group, Institut D’Investigació Sanitària Illes Balears (IdISBa), Carretera de Valldemosa 79, S Building, 1st Floor, 07120 Palma de Mallorca, Spain

## Correction to: Clin Epigenet (2021) 13:150 10.1186/s13148-021-01139-w

Following publication of the original article [1], the authors identified an error in the Funding section.

The section currently read:

This work was supported by the Instituto de la Salud Carlos III Miguel Servet Project (#CP17/00188) and AES 2019 (#I19/01514), the Institut d’Investigació Sanitària Illes Balears (FOLIUM program), the Govern de les Illes Balears (Mar- galida Comas program), the Fundación Francisco Cobos, and the Asociación Española Contra el Cancer.

The section should read:

This work was supported by the Instituto de la Salud Carlos III Miguel Servet Project (#CP17/00188) and AES 2019 (#PI19/01514) co-funded by European Regional Development Fund "A way to make Europe", the Institut d’Investigació Sanitària Illes Balears (FOLIUM program), the Govern de les Illes Balears (Margalida Comas program), the Fundación Francisco Cobos, and the Asociación Española Contra el Cancer.

The original article [1] has been corrected.
